# News and views

**DOI:** 10.1111/iwj.13990

**Published:** 2022-10-28

## 1 MÖLNLYCKE INAUGURATES NEW PLANT AND ACCELERATES TO MEET FUTURE DEMAND FOR SUPPLY AND SUSTAINABILITY

In its pursuit of sustainable growth and to meet future demands for supply and sustainability Mölnlycke has inaugurated a new surgical gloves factory in Kulim, Malaysia. The investment amounts to EUR 50 million and will allow Mölnlycke to substantially increase their production capacity.

The new site is fully equipped for cutting‐edge automated glove production and packing, and Mölnlycke expects to create 400 new job opportunities. Mölnlycke will also increase their production capacity substantially.

The new facility is a major milestone in Mölnlycke's sustainability roadmap “WeCare” and the company's ambition to become a global leader in sustainable healthcare, while being well on the way to decarbonise all Mölnlycke factories, the company is investing in solutions that will lower its CO_2_ emissions also in this plant.

Surgical glove production is energy‐ and water‐intensive and as announced earlier this month, Mölnlycke have partnered with ENGIE, who will be providing energy solutions, one being installing solar panels to power the plant's manufacturing and operations and with Veolia to improve water management. Veolia will be assisting in improving the plant's wastewater management, effectively recycling water and improving the discharge quality. With Veolia's digitalised systems, water consumption is expected to be reduced by up to 50% locally.

In the long term, Mölnlycke is turning its commitment to net zero greenhouse gas (GHG) emissions by 2050 into reality by running its operations in a less resource‐intensive way.

With sustainability as a key aspect of the company's strategy, Mölnlycke is setting out to build a sustainable healthcare manufacturing ecosystem that will effectively meet the industry's demand for high‐quality surgical gloves, with minimal environmental impact.

Zlatko Rihter, CEO of Mölnlycke stated, “There is a lot to be proud of when working at Mölnlycke. When future generations look back at our history, I am convinced they will see this day as a milestone—as we take yet another step on our journey to become a global leader in sustainable healthcare production and supply and a leading employer in the global healthcare sector.”

## 2 ARCH THERAPEUTICS ANNOUNCES FIRST SHIPMENTS OF AC5 ADVANCED WOUND SYSTEM UNDER NEW REIMBURSEMENT SUPPORT PROGRAM

Arch Therapeutics, Inc. a marketer and developer of novel self‐assembling wound care and biosurgical products, confirmed the first shipments of AC5 Advanced Wound System (“AC5”) under the Company's new reimbursement support program (the “Program”). This development represents a significant milestone in the Company's overall commercialisation effort, which management believes will support the accelerated growth and use of AC5 in doctor's offices and wound care clinics.

AC5 is a synthetic self‐assembling wound care product that provides clinicians with multi‐modal support and utility across all phases of wound healing. AC5 was cleared by the Food and Drug Administration (“FDA”) for the management of partial and full‐thickness wounds, such as pressure sores, leg ulcers, diabetic ulcers, and surgical wounds. The Program seeks to leverage recently announced policy changes from the Centers for Medicare and Medicaid Services (“CMS”) for synthetic skin substitutes while the Company is awaiting a response on its HCPCS application for a unique code for AC5. The Program is also intended to facilitate the Company's engagement with payors to advocate for clinically appropriate AC5 coverage and payment policies.

“This is an exciting time for Arch and the launch of the reimbursement support program represents a huge step forward. Not only do CMS's policy changes provide doctors with greater clarity regarding the potential billing and reimbursement pathway for their use of AC5 to treat patients, but the Program also supports our ability to work with payors to develop specific, appropriate AC5 reimbursement policies. Taken together, we expect they will further promote enhanced customer confidence in AC5. In September alone, we expect to invoice more than twice the total number of commercial units shipped year to date. While the total number of units remains relatively modest, we believe the foundation provided by the Program will drive the acceleration of near and long‐term revenue growth opportunities for the Company,” stated Dan Yrigoyen, Vice President of Sales of Arch Therapeutics.

About Arch Therapeutics, Inc. Arch Therapeutics, Inc. is a biotechnology company with a novel approach to stop bleeding (haemostasis), control leaking (sealant) and manage wounds during surgery, trauma, and interventional care. Arch is developing wound care and biosurgical products based on an innovative self‐assembling peptide technology platform with the goal of improving healing outcomes for patients. Arch has received regulatory clearance to market AC5 Advanced Wound System in the United States and AC5 Topical Hemostat in Europe. Arch's development stage product pipeline includes AC5‐G for endoscopic resection of gastrointestinal tumours, AC5‐V for haemostasis during vascular surgery and AC5 Surgical Hemostat for general surgical haemostasis, among others.

## 3 A NEW CLINICAL‐GRADE COLLAGEN MADE FROM DISCARDED BULLFROG SKIN IS LEAPFROGGING FROM THE LAB BENCH TO CLINICS SOON

The collagen is developed by materials scientists from Nanyang Technological University, Singapore (NTU Singapore) in collaboration with Singapore medical technology firm Cuprina Wound Care Solutions (Cuprina), which specialises in developing products that treat chronic wounds, such as those suffered by diabetes patients.

Through NTU's innovation and enterprise company, NTUitive, the patented technology for converting waste bullfrog skin into skin wound healing products has been exclusively licensed to Cuprina for scale‐up and commercial production. If successful, this new product will complement Cuprina's flagship product MEDIFLY, a bio‐dressing made of live, medical‐grade Lucilia cuprina maggots, which is used in Maggot Debridement Therapy.

MEDIFLY is clinically proven to eliminate chronic wound infections and reduce amputation rates caused by wounds, especially in cases associated with diabetic foot ulcers, and is used by hospitals and specialist clinics across Singapore. Chronic wounds affect 1 in 20 patients in Singapore. Coupled with diabetes affecting 1 in 10 patients and a rapidly aging population, the risk of developing a chronic wound is high and demand for affordable chronic wound care is expected to increase.

Currently, 20 million tonnes of fishery by‐products, such as fins, scales, and skins, are discarded every year, and the combined annual consumption of frog flesh and fish is estimated to be around 100 million kilograms. Using this seafood waste to create valuable collagen is a sustainable way to up‐cycle and reduce waste for Singapore, as the country is driving towards its Zero Waste Masterplan and encouraging a circular bioeconomy.

This research and commercialisation effort will help Singapore to achieve its aim of becoming a sustainable, resource‐efficient, and climate‐resilient nation, says Associate Professor Dalton Tay from NTU's School of Materials Science and Engineering, an expert in animal and plant biomass valorization, who developed the innovation.

By using this collagen‐rich marine by‐product as a raw material, the team hopes to reduce wastage and the product cost of pure collagen at scale, so as to bring an affordable but effective wound‐care solution to the public. Earlier this year, Cuprina's joint proposal with NTU to develop and fast‐track the commercialisation of this unique collagen product was announced as one of the winning entries of the Sustainability Open Innovation Challenge organised by Enterprise Singapore.

Mr David Quek, CEO of Cuprina Holdings said partnership with NTU is the perfect example of how cutting‐edge medical products can be developed while simultaneously removing waste from the environment. The 2‐year research and scale‐up partnership include plans for the conduct of clinical trials in local hospitals to validate its safety and efficacy.

## 4 MIMOSA DIAGNOSTICS REBRANDS FOR GROWTH

As MIMOSA Diagnostics enters its full commercialisation and next growth phase you may notice a fresh new logo, colour palette and new curves on its website. MIMOSA Diagnostics recently underwent a brand refresh. The logo, colour palette, typeface, and more have all been given a style evolution.

*“Our technology is evolving. Therefore, our brand required evolving to match. We want to celebrate our transformation from a startup to a growing full service mobile imaging company with this new visual identity. A bright, energetic visual language and image that is both friendly and adaptable.” Dr Karen Cross, CEO*.

The original yellow tone, based on the MIMOSA flower and the light pattern emerging from its device was a testament to the companies' youth and bursting energy they wanted to bring to its customers. Five years down the road, its technology has evolved and grown, and so has its team—“we've tripled our headcount since then”, said Dr Cross. “The flower is blossoming and growing!”

The product team is constantly evolving the MIMOSA technology and platform to make it easier for clinical teams to understand and assess patients. MIMOSA is developing stronger capabilities, enabling teams to capture more data for better clinical insights, and to provide better care, helping save limbs and lives. Vascular compromise is no longer hidden or missed, as MIMOSA makes the invisible, visible!

*“The original bright, energy of the yellow was tweaked to a richer hue—conveying maturation, trust, growth and stability, with an edge of sophistication”. Ethan Fenton, Director Cageless Content*.

MIMOSA wanted to celebrate its transformation from start‐up to a full‐blown mobile medical imaging company with this new visual identity. A bright, energetic imaging solution that is both equitable and accessible.

### 4.1 First impressions matter

The brand refresh started with bidding adieu to its beloved energetic, spectral petals, the energetic child of yellow that brought us to where we are today. “If you didn't know before, our logo, both old and new is based on the MIMOSA flower. While it continues to be so, we blossomed the logo by completing the petals, creating the maturity from the bud to the blossom. Nature does this over time and our product, technology and company are evolving into that mature strong flower that makes you stop and take notice. We spent the last few years developing our deep roots and the foundation of our company and the flower represents the fruits of that labour. Just like our product, we have spent time building a solid foundation, reputation and brand as the go to clinical platform for the assessment of tissue health status.” Concluded Dr Karen Cross, CEO, MIMOSA Diagnostics.

So, there you have it. MIMOSA still have the tenacity of a young start‐up and the energetic enthusiasm of the old logo, but it has grown up. It has become the provider of mature, well‐tested and validated technology changing the lives of patients and clinicians globally.

*For more information about MIMOSA Diagnostics email us at* info@mimosadiagnostics.com.

